# A Rare Case of Renal Failure Caused by T-Cell Prolymphocytic Leukaemia Infiltration

**DOI:** 10.1155/crh/1402078

**Published:** 2025-08-29

**Authors:** Stuart K. Gibson, Thomas Davis, Melisa Vazquez, Swe Htet, Eric Wong

**Affiliations:** ^1^Clinical Haematology, Olivia Newton-John Cancer Wellness and Research Centre, Austin Health, Heidelberg, Victoria, Australia; ^2^Anatomical Pathology, Austin Health, Heidelberg, Victoria, Australia; ^3^Haematology Unit, Grampians Health, Ballarat, Victoria, Australia

**Keywords:** leukaemia, lymphoma, renal failure, T-cell prolymphocytic leukaemia, T-PLL

## Abstract

T-cell prolymphocytic leukaemia (T-PLL) is an aggressive and rare post-thymic T cell malignancy, highly refractory to conventional cytotoxic chemotherapeutics. While extranodal involvement is common, solid organ invasion is rare. We present the case of a 76-year-old man who developed acute renal failure secondary to T-PLL renal infiltration. On day four of his admission, prior to commencing alemtuzumab, his creatinine rose from 133 μmol/L to 390 μmol/L, with anuria. Renal biopsy demonstrated an infiltrate of monomorphic, mononuclear cells positive for a STAT5B mutation, consistent with T-PLL infiltration. He required haemodialysis, but was treated with pulsed methylprednisolone and alemtuzumab, with excellent renal recovery, although remission was not achieved. This case demonstrates that renal leukaemic infiltration must be considered in T-PLL patients with rapidly progressive renal failure, and that solid organ invasion should not contraindicate timely commencement of T-PLL-directed therapy with alemtuzumab.

## 1. Introduction

T-cell prolymphocytic leukaemia (T-PLL) is an aggressive and rare post-thymic T cell malignancy, highly refractory to conventional cytotoxic chemotherapeutics [1]. While the anti-CD52 monoclonal antibody alemtuzumab induces high response rates (> 90%), relapse remains almost inevitable, and median survival is less than two years [2, 3].

Clinicopathological understanding of T-PLL is limited by rarity; evidence on presenting features is collated from several small case series [4]. Most patients present with hepatosplenomegaly, generalised non-bulky lymphadenopathy and elevated leucocyte count (≥ 100 × 10^9^/L). Extranodal involvement is common, manifesting as rashes, peripheral oedema and serosal effusions. Solid organ invasion including renal infiltration is exceptionally rare [5].

## 2. Case Presentation

We present the case of a 76-year-old male with T-PLL who developed acute renal failure secondary to leukaemic kidney infiltration. He was diagnosed with T-PLL after presenting with fatigue, weight loss and rapidly rising lymphocytosis. A bone marrow biopsy demonstrated a mildly hypercellular marrow with a moderate excess of abnormal mature lymphocytes, some with notable cytoplasmic blebbing and nucleoli, with an aberrant immunophenotype by flow cytometry (CD2+, CD3+, CD4+, CD5+ (bright), CD7+, CD8−, CD16−, CD56− and CD57−). Cytogenetics revealed an inv(14) (q11.2q32), the chromosomal abnormality seen in 80% of T-PLL involving the *TRAID* and *TCL1A* genes. PET-CT found widespread lymphadenopathy and no extranodal disease. His medical history included hypertension, type two diabetes mellitus and chronic kidney disease with microalbuminuria.

The patient was admitted and planned for alemtuzumab. He was clinically well, with lymphocytes of 90.4 × 10^9^/L and serum creatinine (Cr) of 133 μmol/L (his baseline). On day four of his admission, prior to commencement of T-PLL-directed therapy, his Cr sharply rose to 390 μmol/L. He was euvolaemic, with no oedema, and oligo-anuric. CT of the urinary tract showed no hydronephrosis or calculus. Microscopy of a catheter specimen of urine demonstrated leukocytes and erythrocytes of > 500 × 10^6^/L, with no organisms cultured.

**Figure 1:**
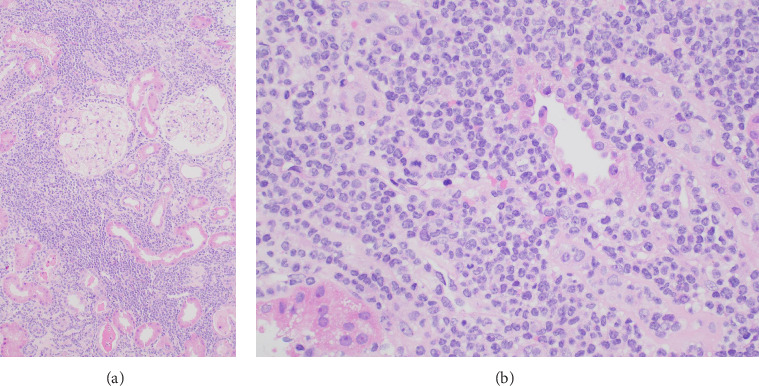
Haematoxylin and eosin (H&E) staining from the renal biopsy. H&E ×100 (a): leukaemia cells infiltrating the renal cortex, surrounding glomeruli and pushing apart tubules, which have features of acute tubular injury. H&E ×400 (b): dense infiltrate of monomorphic, small to medium mononuclear cells with mild nuclear atypia including indented nuclear outlines. Occasional mitoses are also present. Notably, features of crescentic glomerulonephritis are not present.

**Figure 2:**
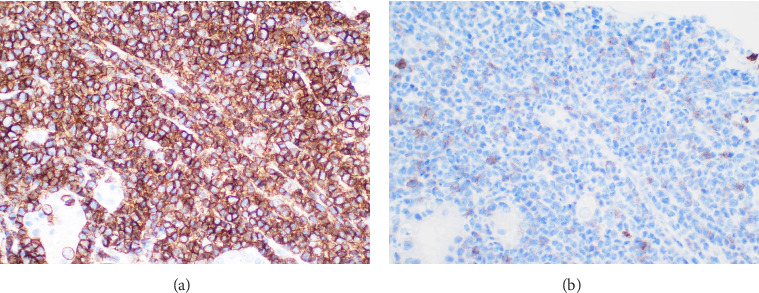
CD4 and CD8 immunohistochemistry from the renal biopsy. CD4 (a): strong and diffuse CD4 expression by immunohistochemistry. CD8 (b): patchy and weak CD8 expression by immunohistochemistry. These findings are consistent with a T-PLL phenotype and concordant with bone marrow flow cytometry.

With Cr reaching 507 μmol/L that evening, after discussion with nephrology, intravenous methylprednisolone 500 mg and rasburicase 6 mg were administered. This methylprednisolone dose was chosen to cover possible rapidly progressive glomerulonephritis (RPGN), considering the urinalysis findings. A haemodialysis catheter was inserted, and renal biopsy was performed on day six, which demonstrated a dense infiltrate of monomorphic, mononuclear cells within the interstitium of the cortex and corticomedullary junction (Figure 1, in addition to acute tubular injury and background fibrotic changes), without features of RPGN. The immunoprofile of the infiltrate (CD3+ and CD4+ with very weak CD8 co-expression, demonstrated in Figure 2) was consistent with a T-PLL phenotype and concordant with bone marrow flow cytometry. Next-generation sequencing from the biopsy demonstrated a STAT5B mutation, further demonstrating the clonal nature of the infiltrate.

Although the patient's urine output improved with methylprednisolone, his Cr continued to rise, peaking at 732 μmol/L, and he required one session of haemodialysis. Treatment of his T-PLL was initiated with alemtuzumab thrice weekly. His renal indices improved, with his Cr recovering to 148 μmol/L 9 days after methylprednisolone was commenced. He was discharged to his local haematologist for continuation of alemtuzumab infusions. While peripheral blood and bone marrow cytoreduction was initially achieved, alemtuzumab therapy was complicated by cytomegalovirus reactivation which was treated with valganciclovir, leading to profound cytopenias. Repeat bone marrow assessment was markedly hypocellular with flow cytometric evidence of residual T-PLL, and alemtuzumab was ultimately suspended due to ongoing infectious complications and cytopenias. Complete remission was never achieved. One year later, the patient had a progressive marked lymphocytosis with associated severe cytopenias, and he had been linked with hospice services.

## 3. Discussion

This case was remarkable for several reasons. The identification of renal T-PLL infiltration as the culprit for rapidly progressive renal failure was surprising. Within haematolymphoid neoplasms, infiltration of kidney parenchyma is not uncommon, with 37.6% of lymphoma patients showing evidence of renal involvement in autopsy in the largest case series [6]. However, this infiltration is usually subclinical and much more associated with B-cell malignancies. Rapidly progressive renal dysfunction secondary to mature T-cell leukaemic infiltration is rare. Although other differentials were considered, including acute interstitial nephritis and RPGN, the infiltrate's homogeneity and positive STAT5 status make T-PLL infiltration by far the most likely diagnosis. External expert review concurred with this impression.

Only one other case of renal T-PLL infiltration has been reported, by Reuben et al. [5]. For their patient, renal failure (requiring haemodialysis) occurred after 12 weeks of alemtuzumab. After renal biopsy showed T-PLL infiltration, the patient was treated with cyclophosphamide and dexamethasone, but no renal recovery was observed and palliation was pursued. Our patient, however, responded rapidly to pulsed methylprednisolone and alemtuzumab, with normalisation of his renal function. This discrepancy may reflect the aggressive nature of alemtuzumab-refractory T-PLL, but also suggests that in cases of T-PLL solid organ infiltration, treatment with alemtuzumab may improve organ function and should not in itself be considered a contraindication to definitive therapy. Interestingly, both patients had a background of CKDIII; it is possible that clinically significant T-PLL renal infiltration may be more likely to manifest as renal failure in patients with poor renal reserve.

In conclusion, this case demonstrates that direct renal leukaemic infiltration should be considered in patients with T-PLL with rapidly progressive renal failure and demonstrates the importance of prompt commencement of T-PLL-directed therapy with alemtuzumab to improve organ function.

## Data Availability

The data that support the findings of this study are available from the corresponding author, SG, upon reasonable request.

## References

[1] Jain P., Aoki E., Keating M. (2017). Characteristics, Outcomes, Prognostic Factors and Treatment of Patients with T-Cell Prolymphocytic Leukemia (T-PLL). *Annals of Oncology*.

[2] Dholaria B. R., Ayala E., Sokol L. (2018). Allogeneic Hematopoietic Cell Transplantation in T-Cell Prolymphocytic Leukemia: A Single-Center Experience. *Leukemia Research*.

[3] Dearden C. E. (2006). T-Cell Prolymphocytic Leukemia. *Medical Oncology*.

[4] Matutes E., Brito-Babapulle V., Swansbury J. (1991). Clinical and Laboratory Features of 78 Cases of T-Prolymphocytic Leukemia. *Blood*.

[5] Reuben S., Saleh N., Kardan A. (2016). Direct Renal Infiltration of T-Cell Prolymphocytic Leukemia. *American Journal of Kidney Diseases*.

[6] Miyake O., Namiki M., Sonoda T., Kitamura H. (1987). Secondary Involvement of Genitourinary Organs in Malignant Lymphoma. *Urologia Internationalis*.

